# Parental acceptance of ECG screening for school-aged children: influencing factors and recommendations

**DOI:** 10.3389/fpubh.2025.1704354

**Published:** 2025-12-17

**Authors:** Khaled Elenizi

**Affiliations:** Department of Internal Medicine, College of Medicine, Prince Sattam bin Abdulaziz University, Al-Kharj, Saudi Arabia

**Keywords:** electrocardiogram, health education, school-age, prevention, parents

## Abstract

**Introduction:**

ECG screening is vital for the early identification of genetic, congenital, and other cardiovascular conditions in young individuals, potentially reducing the risk of sudden cardiac arrest (SCA). This study aims to evaluate parental knowledge and attitudes toward ECG screening and to identify the factors influencing their acceptance or reluctance.

**Method:**

A cross-sectional survey was conducted involving 1,137 parents of school-aged children in Al-Kharj, Saudi Arabia. The questionnaire gathered demographic information, assessed the level of awareness regarding inherited cardiac diseases, and evaluated parental attitudes and perceptions toward ECG screening for children.

**Results:**

The findings revealed a significant lack of understanding regarding the effectiveness of ECG in diagnosing inherited cardiac diseases. Only 26.3% of parents were familiar with the concept of ECG screening, while 73.7% were unaware of it. Furthermore, over one-third (35.7%) had no knowledge of ECG, and 48.1% had only heard of it. Notably, 25.6% opposed ECG screening for their children, primarily due to confidentiality concerns (24.1%). Despite these concerns, 46.4% acknowledged the potential benefits of ECG screening in preventing serious cardiac diseases, and 23.4% trusted the test’s reliability. Additionally, 42.9% of respondents felt that schools or general practitioners should offer more information regarding ECG testing.

**Conclusion:**

The study highlights a critical lack of awareness and understanding of ECG screening among parents, emphasizing the need for enhanced educational initiatives and public awareness campaigns. By improving parental knowledge, acceptance of cardiovascular screening in youth may increase, ultimately contributing to better health outcomes.

## Rationale

Following the observation of a significant number of parents declining ECG screening for their children as part of the Al-Kharj’s Screening Program for Identifying ECG Anomalies in School-Age Children (ASPIRE), we conducted this survey to explore parental knowledge, acceptance, and perspectives on ECG screening and its role in preventing sudden cardiac arrest (SCA) in youth. The ASPIRE program is a region-wide school-based cardiovascular screening initiative in Al-Kharj, Saudi Arabia that includes ECG to detect early cardiac abnormalities in school age children.

## Introduction

Sudden cardiac arrest (SCA) in young individuals is linked to both structural and electrical cardiac abnormalities. An electrocardiogram (ECG) can help identify a significant number of those affected. For instance, hypertrophic cardiomyopathy (HCM) displays abnormal ECG findings in up to 95% of cases, while arrhythmogenic right ventricular cardiomyopathy shows abnormalities in approximately 80% of cases. Long QT syndrome (LQTS) can be detected in 85–90% of cases via ECG. Other conditions associated with SCA include Brugada syndrome, Wolff–Parkinson–White syndrome (which is characterized by delta waves), catecholaminergic polymorphic ventricular tachycardia, coronary artery anomalies, congenital heart defects, myocarditis, and Marfan syndrome. It’s noteworthy that about 10 to 15% of cases classified as sudden infant death syndrome (SIDS) are actually linked to channelopathies (1–3).

Cardiovascular health is a critical component of a child’s overall well-being, and early detection of hereditary or congenital heart conditions is pivotal in preventing severe outcomes. ECG screening, a non-invasive method, is essential for identifying potential cardiac abnormalities. However, parental knowledge and acceptance of ECG screening for school-aged children remain understudied. This study aims to evaluate parental knowledge, attitudes, and concerns regarding ECG screening for their children, while identifying factors that influence their acceptance or hesitation.

Although this study focuses on school-aged children, ECG screening is equally important in preschool populations. Many genetic and congenital cardiac conditions, such as long QT syndrome, Wolff–Parkinson–White syndrome, cardiomyopathies, and congenital conduction abnormalities, may be present from infancy or early childhood but remain clinically silent until later years. Early ECG screening in preschool children can enable timely diagnosis, prevent life-threatening arrhythmias, and guide follow-up before children enter more physically active school environments. Evidence has shown that detecting electrical abnormalities before age six significantly improves long-term outcomes and reduces the risk of delayed diagnosis (4–6).

Evidence suggests that increasing awareness about health risks and injuries in youth can positively influence parental attitudes and behavior. For instance, a study by Bloodgood et al. (7) examined awareness, knowledge, and perceptions of concussion among American youth athletes and their parents, finding that parents were highly receptive to health education. Similarly, Kennedy et al. (8) emphasized the importance of healthcare providers addressing knowledge gaps through educational materials targeting both parents and children. The aim of this study was to evaluate parental knowledge of the utility of cardiovascular screening in youth and to compare knowledge levels across varying parental educational backgrounds.

## Methods

### Study design

A cross-sectional survey was conducted in the Al-Kharj region, Saudi Arabia targeting parents of school-aged children across all schools in the region. It was conducted in the month of December 2024. The questionnaire, designed in Arabic, comprised demographic data, educational and socioeconomic status, knowledge of inherited cardiac diseases, awareness of ECG, and attitudes toward ECG screening for children. The survey was distributed digitally, ensuring accessibility to a broad demographic. Data were anonymized and analyzed using descriptive and inferential statistics to determine trends and correlations.

### Survey development

A survey was developed to evaluate parental knowledge, experiences, and attitudes related to ECG screening, sudden cardiac arrest (SCA), and cardiovascular risk in youth. The survey was administered via Google Forms and assessed various parental characteristics, including age, gender, educational level, and number of children eligible for ECG screening.

The survey also captured youth characteristics, including age and gender. Parental knowledge of ECG screening was assessed through questions. Additionally, we evaluated parental awareness of the importance of ECG screening, attitudes toward its implementation, and willingness to participate in preventive programs through directed survey items and optional open-ended responses.

The questionnaire was self-developed based on a literature review and the aims of the study. Content validity was ensured through review by three experts in cardiology and public health. A pilot test was performed with 20 parents to confirm clarity and usability. Internal-consistency reliability was acceptable, with Cronbach’s alpha values of 0.78 for knowledge items and 0.82 for attitude items.

### Population and data collection

The survey was administered electronically via Google Forms and distributed through the participating schools to all parents in all grades for the Al-Kharj ECG screening program. Completion of the survey was voluntary, and parents were invited to participate regardless of whether they intended to consent to ECG testing for their child. An introductory section preceded the questionnaire, explaining the study purpose, ensuring confidentiality, and emphasizing that participation was entirely voluntary.

To ensure data completeness and accuracy, only surveys where 100% of the questions were answered were included in the analysis. Surveys with incomplete responses were excluded entirely. This strict inclusion criterion applied both to overall survey completion and to responses regarding individual children, ensuring that all data collected were comprehensive and reliable.

### Sampling considerations and potential bias

The questionnaire was disseminated to all parents in the participating schools where ECG screening was planned, and survey participation was entirely voluntary. Parents were invited to complete the questionnaire regardless of whether they intended to consent to ECG screening for their child; therefore, respondents represent parents who elected to participate in the survey rather than a cohort preselected by screening approval. As parents who declined to complete the survey were not captured, differential characteristics between respondents and non-respondents cannot be excluded. This introduces the potential for non-response (volunteer) bias, which should be considered when interpreting parental attitudes and the reported reasons for hesitancy toward ECG screening.

### Outcome measures

The primary outcome was parental knowledge regarding the utility of ECG screening for youth. This knowledge was defined as understanding that ECG screening can discover cardiac diseases. Comparisons were made between parents’ education levels and family histories of cardiac diseases with knowledge of cardiac diseases and likelihood to approve of ECGs.

### Statistical analysis

Data were analyzed using the SPSS V.26 (Statistical Package for the Social Sciences) software. Continuous data were presented as mean ± SD (standard deviation) as the data were normally distributed and categorical data as numbers with percentages. A clustered bar graph was used to demonstrate the causes of hesitancy toward performing ECG. Next, the association between the parent’s education level and their knowledge about cardiac diseases and ECG monitoring was assessed using Chi-square testing. Furthermore, the level of knowledge was correlated with the likelihood of accepting ECG using Chi-square testing. The relation between positive family history and knowledge about ECG screening was analyzed by Chi-square testing. Throughout the analysis, a *p*-value of less than 0.05 was considered statistically significant.

## Results

### Parents’ characteristics

A total of 1,137 parents participated in the survey (Table 1), with the majority being mothers, who constituted 73% of the sample, while fathers made up 26.6% and other relations represented only 0.4%. The average age of the parents was 40.2 years, with a standard deviation of 8, indicating that most participants are in middle age. In terms of marital status, a significant proportion (92.6%) were married, while 4.3% were separated or divorced, and 3.1% were widowed. The educational background of the parents showed a diverse range of qualifications; 38.9% held a Bachelor’s degree, 36.1% completed high school, 21.1% had less than a high school education, and 4% pursued postgraduate studies, suggesting a predominantly educated population. Economically, most families reported modest incomes, with over a third (36.8%) earning less than 5,000 SAR per month, 29.6% earning between 5,000 and 10,000 SAR, and only 4.7% earning over 20,000 SAR monthly.

**Table 1:** Parent and child information.

Parent and child information		
Guardian	Father	303 (26.6%)
	Mother	830 (73%)
	Other	4 (0.4%)
The mean age of guardian ±SD		40.2 ± 8
Marital status	Married	1,053 (92.6%)
	Separated/divorced	49 (4.3%)
	Widow	35 (3.1%)
Educational level	Less than high school	240 (21.1%)
	High school	410 (36.1%)
	Bachelor’s degree	442 (38.9%)
	Postgraduate degree	45 (4%)
Monthly salary	Less than 5,000 SAR	418 (36.8%)
	From 5,000 to 10,000 SAR	337 (29.6%)
	From 10,000 to 15,000 SAR	217 (19.1%)
	From 15,000 to 20,000 SAR	111 (9.8%)
	More than 20,000 SAR	54 (4.7%)
The mean of students’ age ± SD		10.4 ± 2.9
Students’ gender	Male	467 (41.1%)
	Female	670 (58.9%)
Number of students in each grade	Kindergarten	14 (1.2%)
	Grade 1	144 (12.7%)
	Grade 2	136 (12%)
	Grade 3	132 (11.6%)
	Grade 4	119 (10.5%)
	Grade 5	125 (11%)
	Grade 6	141 (12.4%)
	Grade 7	79 (6.9%)
	Grade 8	85 (7.5%)
	Grade 9	61 (5.4%)
	Grade 10	29 (2.6%)
	Grade 11	44 (3.9%)
	Grade 12	24 (3.9%)
Family history of cardiac diseases	Yes	226 (19.9%)
	No	911 (80.1%)

### Students’ characteristics

The average age of the students was 10.4 years, with a standard deviation of 2.9. Female students represented 58.9% of the sample, while male students comprised 41.1%. When examined by grade level, there is a higher concentration of students in the lower grades. For instance, Grades 1 through 6 together account for the majority of students, with Grade 1 having the highest representation at 12.7%. In contrast, the number of students decreases in the higher grades, with only 5.4% in Grade 9 and just 3.9% in Grade 12.

### Family history of cardiac diseases

The majority of families, 80.1%, report no history of cardiac diseases, while 19.9% have a positive family history of such conditions.

### Knowledge about inherited cardiac diseases

The survey results (Table 2) revealed varying levels of awareness about inherited cardiac diseases among the respondents. Approximately 12% of respondents had no knowledge about these conditions, while 36.9% reported having little knowledge. The majority, 44.4%, indicated moderate knowledge, and only 6.7% had extensive knowledge about inherited cardiac diseases.

**Table 2:** Survey questions and answers.

Survey questions		
Knowledge about inherited cardiac diseases *N* = 1,137	No knowledge at all	137 (12%)
	Little knowledge	419 (36.9%)
	Moderate knowledge	505 (44.4%)
	Extensive knowledge	76 (6.7%)
Have you or any of your family discussed with your GP cardiac health and inherited diseases	No, and I know nothing about the	458 (40.3%)
	risk	554 (48.7%)
	No, but I know about the risk	80 (7%)
	Yes, occasionally	45 (4%)
	Yes, frequently (extensively)	
Have you heard about ECG screening	Yes	299 (26.3%)
	No	838 (73.7%)
What are the symptoms of inherited cardiac diseases (you may select multiple options)	Sudden death	483 (42.5%)
	Shortness of breath	560 (49.3%)
	Palpitations	581 (51.1%)
	Syncope	342 (30.1%)
	No symptoms	65 (5.7%)
	Not sure	152 (13.4%)
	I do not know	269 (23%)
How worried are you about inherited cardiac diseases	Not worried at all	271 (23.8%)
	Slightly worried	269 (23.7%)
	Moderately worried	289 (25.4%)
	Extremely worried	308 (27.1%)
How would you rate your knowledge of ECG?	No knowledge at all	406 (35.7%)
	Just heard about it	547 (48.1%)
	I have basic knowledge	114 (10%)
	Very knowledgeable about the aim and procedure	70 (6.2%)
How effective do you think ECG is?	Not effective at all	41 (3.6%)
	Slightly effective	96 (8.4%)
	Moderately effective	127 (11.2%)
	Highly effective	361 (31.8%)
	I am not sure	479 (42.1%)
Where have you heard about ECG?	Social media	223 (19.6%)
	Articles	45 (4%)
	School or social campaigns	135 (13.5%)
	GP or health practitioner	153 (13.5%)
	Family or friends	113 (9.9%)
	Haven’t heard about ECG	468 (41.2%)
Would you be willing to have an ECG performed on your child?	Never	164 (14.4%)
	Unlikely	127 (11.2%)
	Neutral	205 (18%)
	Likely	275 (24.2%)
	Very likely	366 (32.2%)
If you are hesitant about having an ECG performed on your child, what would be the reason?	Financial concerns	153 (13.5%)
	Fear of discovering an unknown disease	149 (13.1%)
	Lack of trust in the test’s accuracy	102 (9%)
	Concern about confidentiality	274 (24.1%)
	Other reasons	359 (31.6%)
Do you agree with the following statements about ECG in children?	ECG screening might help in the prevention of serious cardiac diseases in children (yes)	527 (46.4%)
	ECG is trustworthy (yes)	266 (23.4%)
	I am worried about unnecessary anxiety if my child undergoes an ECG (yes)	253 (22.3%)
	ECG test is costly (yes)	135 (11.9%)
	My child’s school or GP should provide more information about ECG testing (yes)	488 (42.9%)
What are your concerns (if any) about ECG testing in children?	Financial constraints	291 (25.6%)
	Confidentiality of the test results	164 (14.4%)
	Psychological stress for me and my family if the results are positive	598 (52.6%)
	Lack of information from the GP	339 (29.8%)
	Unnecessary medical follow up	258 (22.7%)
If the test if provided for free, will you change your opinion about it?	No, it will not affect my opinion	319 (28.1%)
	Yes, I would perform the test	547 (48.1%)
	I am not sure	271 (23.8%)
Do you believe ECG screening should be mandatory for school children?	No, I do not think it is necessary	171 (15%)
	Yes, but only for children with a family history of cardiac diseases	323 (28.4%)
	Yes, to all school children	509 (44.8%)
	I am not sure	134 (11.8%)
How likely are you to share your family history of cardiac diseases with your GP if doing so could help decrease your risk?	Very unlikely	85 (7.5%)
	Unlikely	67 (5.9%)
	Neutral	155 (13.6%)
	Likely	405 (35.6%)
	Very likely	425 (37.4%)

### Discussions about cardiac health and inherited diseases with GPs

A significant number of respondents (40.3%) had never discussed cardiac health and inherited diseases with their general practitioner (GP) and were unaware of the risks. However, 48.7% acknowledged the risks despite not having such discussions. Only 7% reported occasional discussions, and 4% stated frequent or extensive discussions with their GP.

### Awareness of ECG screening

Awareness of ECG screening was low among the respondents, with only 26.3% indicating familiarity with the concept. A majority, 73.7%, had not heard about ECG screening.

### Concern about inherited cardiac diseases

The level of concern about inherited cardiac diseases varied among the respondents. While 23.8% were not worried at all, 23.7% reported being slightly worried. A quarter (25.4%) were moderately worried, and 27.1% expressed extreme worry about these conditions.

### Knowledge about ECG

Knowledge about ECG among respondents was limited. Over a third (35.7%) admitted to having no knowledge at all, and 48.1% stated they had only heard about it. Meanwhile, 10% reported basic knowledge, and only 6.2% considered themselves very knowledgeable about the procedure and its purpose.

### Perceived effectiveness of ECG

A small proportion (3.6%) believed ECG was not effective at all, and 8.4% thought it was slightly effective. Meanwhile, 11.2% deemed it moderately effective, and 31.8% considered it highly effective. However, 42.1% of respondents were unsure about ECG’s effectiveness.

### Sources of information about ECG

Respondents cited various sources of information about ECG. Social media was the most common (19.6%), followed by GPs or health practitioners (13.5%) and school or social campaigns (11.9%). Family or friends contributed to 9.9% of awareness, while 41.2% of respondents admitted they had never heard about ECG.

### Willingness to have an ECG performed on children

When asked about their willingness to have an ECG performed on their children, 14.4% of respondents said they would never consider it, and 11.2% were unlikely to agree. About 18% were neutral, while 24.2% were likely, and 32.2% were very likely to consent to ECG screening for their children.

### Hesitancy about ECG testing for children

**Figure 1:**
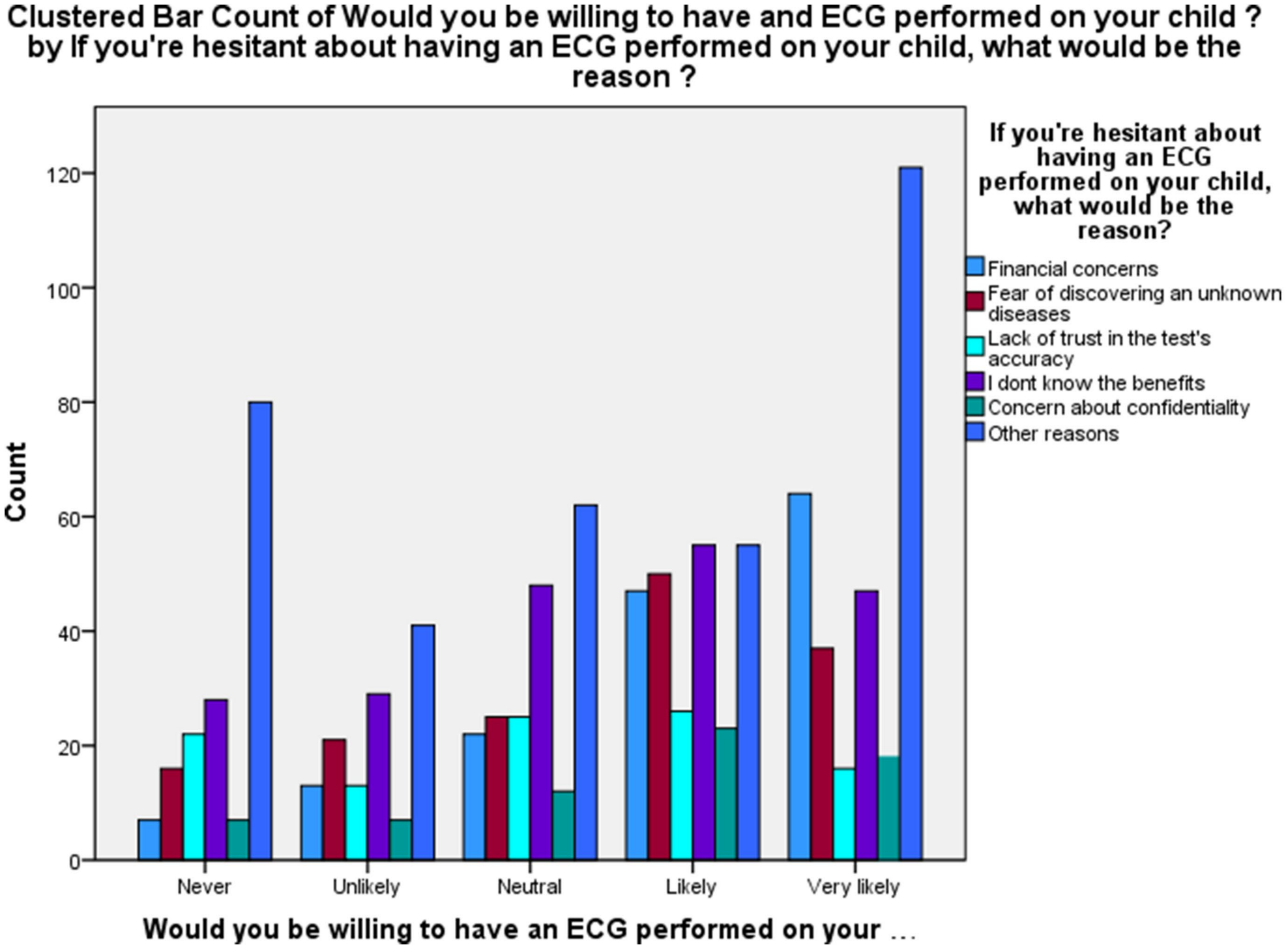
Hesitancy about ECG testing for children.

The primary reasons for hesitancy about ECG testing for children included concern about confidentiality (24.1%) and other unspecified reasons (31.6%). Financial concerns were cited by 13.5%, fear of discovering an unknown disease by 13.1%, and lack of trust in test accuracy by 9% (Figure 1).

### Agreement with statements about ECG testing

Respondents largely agreed that ECG screening could help prevent serious cardiac diseases in children (46.4%) and that the test is trustworthy (23.4%). However, 22.3% expressed concern about unnecessary anxiety, and 11.9% felt the test was costly. A significant number (42.9%) believed schools or GPs should provide more information about ECG testing.

### Concerns about ECG testing in children

Concerns about ECG testing included psychological stress (52.6%), financial constraints (25.6%), and lack of information from GPs (29.8%). Additionally, 14.4% raised concerns about confidentiality, and 22.7% worried about unnecessary medical follow-ups.

### Impact of free ECG testing

When asked if providing the test for free would influence their decision, 48.1% of respondents said they would then agree to the test, while 28.1% stated it would not change their opinion. Meanwhile, 23.8% were unsure about the impact of free testing.

### Mandatory ECG screening for schoolchildren

Regarding mandatory ECG screening for schoolchildren, 44.8% supported screening for all children, and 28.4% agreed it should only be mandatory for those with a family history of cardiac diseases. Meanwhile, 15% believed it was unnecessary, and 11.8% were unsure.

### Sharing family history with GPs

Respondents showed a positive attitude toward sharing family history of cardiac diseases with their GP if it could reduce risks. While 7.5% were very unlikely and 5.9% were unlikely to share, 35.6% were likely, and 37.4% were very likely to share such information.

### Parental education level and knowledge of cardiac diseases and ECG screening

Higher education levels correlate with better knowledge of inherited cardiac conditions. For example, 33.3% of individuals with postgraduate education show good knowledge, compared to only 2.9% of those with less than high school education (Supplementary Figure S1). The chi-square test shows a significant association (*p* < 0.001) (Supplementary Figure S2). Additionally, Knowledge about ECG screening is higher among individuals with postgraduate education (37.8%) compared to those with less than high school education (27.5%). A majority (72.5%) of those with less education report no knowledge (Supplementary Figure S3). The chi-square test confirms a significant association (*p* = 0.038) (Supplementary Figure S4).

### Parental knowledge and likelihood to approve ECG

Individuals with higher education levels are more likely to approve ECG screening. For example, 53.3% of those with postgraduate education are very likely to approve, compared to 23.3% of individuals with less than high school education. In contrast, 17.9% of those with less than high school education would never approve ECG screening, dropping to 11.1% among postgraduates (Supplementary Figure S5). The chi-square test confirms a significant association (*p* < 0.001), with a Pearson chi-square value of 36.022 and a likelihood ratio of 36.612 (Supplementary Figure S6). These findings highlight the impact of education on fostering positive attitudes toward preventive healthcare.

### Family history and knowledge about cardiac diseases and ECG screening

Individuals with a positive family history show higher knowledge levels about cardiac diseases, with a significant association confirmed by the chi-square test (*p* < 0.001) (Supplementary Figures S7, S8). No significant association was found between family history of cardiac disease and knowledge about ECG screening (*p* = 0.454) (Supplementary Figures S9, S10).

### Symptoms thought to be related to inherited cardiac diseases

**Figure 2:**
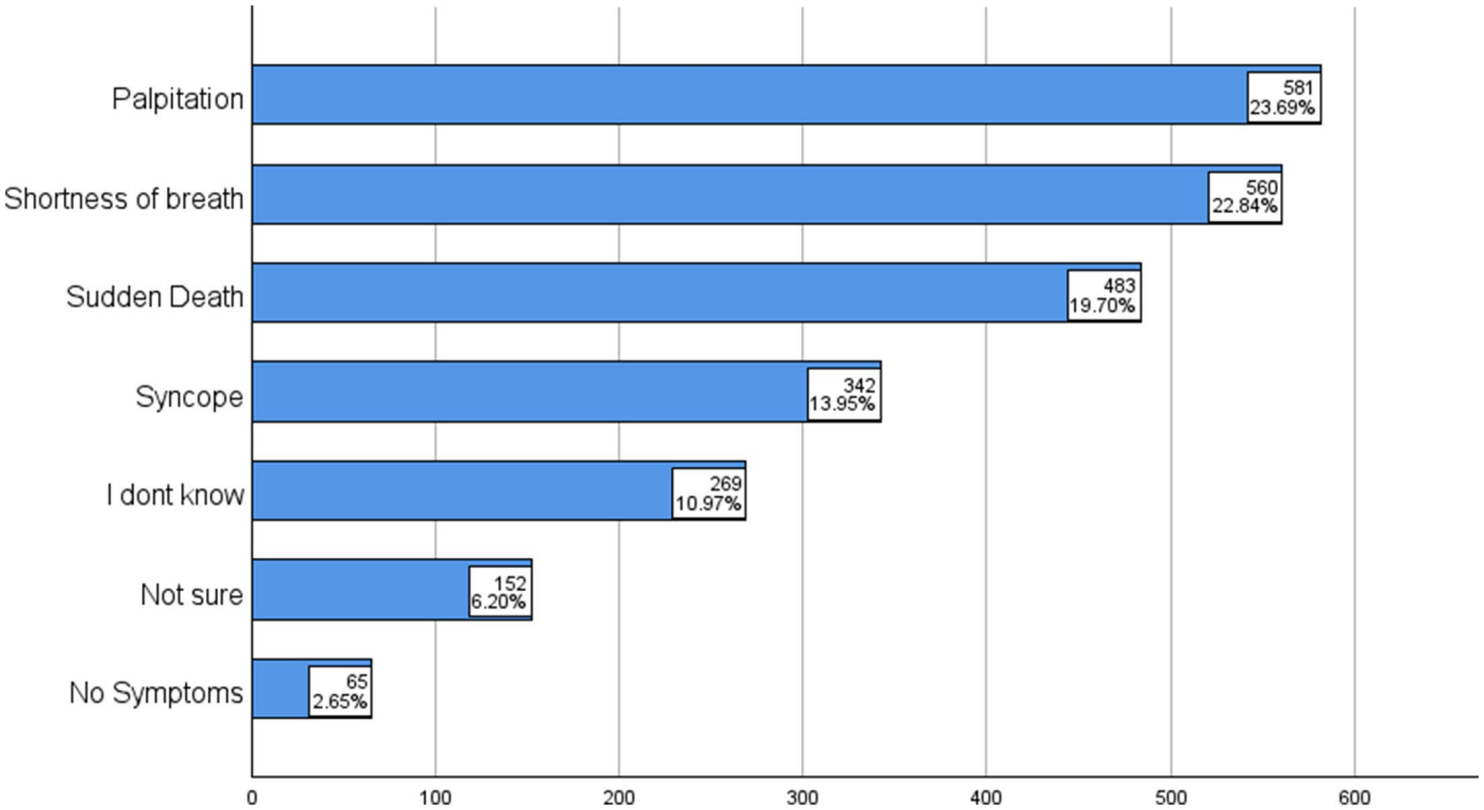
Symptoms thought to be related to inherited cardiac diseases.

Parents’ awareness of symptoms varied considerably. Shortness of breath (49.3%) and palpitations (51.1%) were the symptoms most commonly selected, followed by sudden death (42.5%) and syncope (30.1%). However, a substantial proportion of parents were either uncertain (13.4%) or unaware (23%), indicating a notable knowledge gap in recognizing clinical warning signs (Figure 2).

### How did you hear about ECG

**Figure 3:**
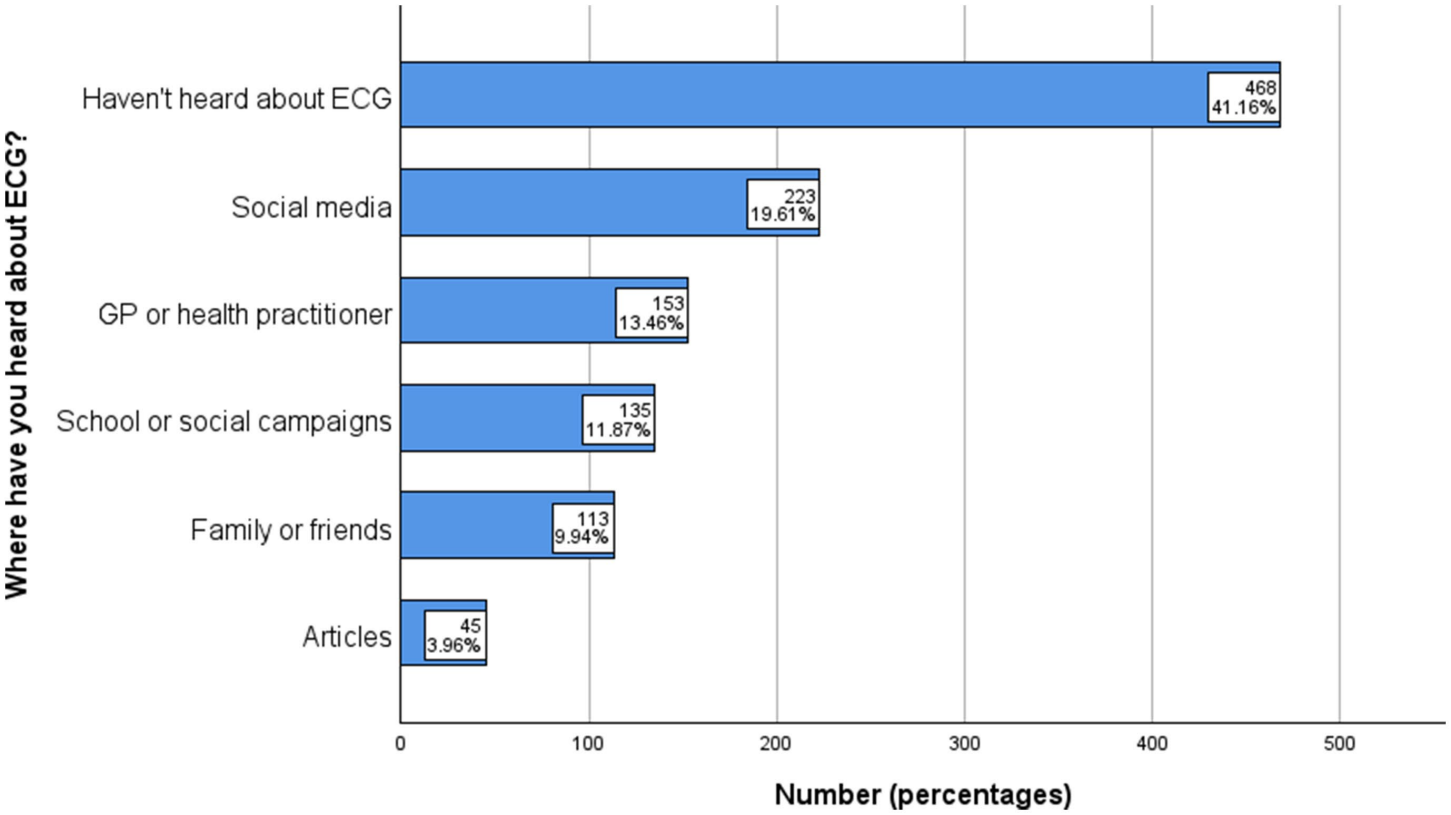
How did you hear about ECG.

The chart reveals significant gaps in ECG screening awareness, with 41.16% of respondents unaware of it. Social media (19.61%) and medical practitioners (13.46%) were key sources of information, while school campaigns (11.87%) and personal networks (9.94%) played smaller roles. News articles (3.96%) had minimal impact. These findings emphasize the need for multi-channel strategies, prioritizing social media, healthcare providers, and educational campaigns (Figure 3).

## Discussion

The American Heart Association (AHA) and the European Society of Cardiology (ESC) advocate for cardiovascular screening of asymptomatic young athletes, citing ethical, legal, and medical reasons. However, the AHA’s opposition to mandatory national ECG screening in the U.S. has ignited criticism (9–11). This raises a key question: should screening be limited to competitive athletes when other young people may also be at risk? Broadening screening to the entire youth population promotes equity and ensures early detection of serious cardiac conditions for all. Data indicate that the incidence of sudden cardiac death in young people in the general populations is often higher than in competitive athletes (12, 13). For instance, reported rates of out-of-hospital cardiac arrest are 6.4 per 100,000 person-years in one pediatric group (14) and 3.76 per 100,000 person-years in Denmark’s youth population (15).

Screening programs for children aged 1 to 12 are rare, with the 1973 Japanese School Health Law being a notable example that mandated cardiovascular screening, including modified ECGs and physical exams, for students in the first, seventh, and tenth grades. While this initiative screened thousands of children, it primarily identified minor cardiovascular abnormalities without underlying heart disease in only 2 to 3% of cases, suggesting that significant cardiac conditions may be low in this age group. Nevertheless, screening remains valuable for early detection, highlighting the need to refine protocols to better target higher-risk individuals (16, 17).

Comprehensive assessments that include personal and family history along with physical exams are useful but often have high false-negative rates for detecting cardiovascular issues linked to sudden death in athletes (18), underscoring the importance of supplementary tools like ECGs (19, 20). These are crucial for accurately identifying conditions such as ion channelopathies and Wolff–Parkinson–White syndrome, where physical exams may yield unremarkable results.

To implement ECG screening programs for school-aged children, it is essential to measure parental acceptance. However, studies on parental knowledge and acceptance of such screening are limited (21). This study found that only 26.3% of parents were familiar with ECG screening, while 73.7% were unaware of it. Additionally, 35.7% had no knowledge of ECG, and 48.1% had only heard of it. Opposition to screening was 25.6%, primarily due to concerns about confidentiality. Despite these concerns, 46.4% recognize the potential of ECG screening to prevent serious cardiac diseases, while 23.4% trusted its reliability. Furthermore, 42.9% believe that schools or general practitioners should provide more information about ECG testing. Personal factors, such as a family history of cardiac disease, significantly impact health knowledge; those with such a history are more likely to seek information, whereas others may be less aware of the associated risks. Tailored education is crucial for bridging this gap and promoting widespread awareness of the risks and prevention of cardiac diseases.

The importance of early ECG evaluation extends beyond school-aged children. Several studies have demonstrated that cardiac electrical abnormalities may be identifiable in early childhood, including the preschool years, even in the absence of symptoms. Early detection plays a crucial role in preventing adverse events, particularly in conditions such as congenital long QT syndrome, Brugada syndrome, and pre-excitation syndromes, which may present with sudden arrest as the first manifestation. Findings from Japan and Italy show that cardiovascular screening beginning before formal schooling enables detection of congenital abnormalities at a stage when interventions are most effective (22–24). These early-screening models highlight the potential value of ECG assessment in preschool children, especially in populations where parental awareness is limited, as observed in this study.

Parents expressed hesitation toward ECG testing for several reasons, as illustrated in Figure 1, with the most frequent being concern about result confidentiality (24.1%), followed by fear of uncovering an undiagnosed condition (13.1%), financial burden (13.5%), and uncertainty about the test’s accuracy (9%). Comparable findings have been described in studies of adolescent and parental health behavior, where limited privacy assurances and poor communication reduced participation in screening programs (25, 26).

Parental acceptance of preventive health measures generally improves with adequate education and reduced costs; therefore, increasing awareness through community campaigns, educational initiatives in schools, and engagement with healthcare providers is essential. Addressing financial concerns through government-sponsored free screening programs can enhance participation rates, while clear communication about the benefits and limitations of ECG screening may alleviate fears of overdiagnosis and unnecessary interventions. A clear recommendation from a trusted physician remains one of the strongest predictors of parental consent for preventive procedures (27). Economic constraints also play a key role, and previous work has shown that removing or subsidizing screening costs markedly increases participation (28). Overall, these data indicate that hesitancy reflects modifiable barriers such as limited information, financial burden, and confidentiality concerns rather than resistance to preventive care itself.

To improve participation in our region, several practical and evidence-based actions are proposed. First, integrating short awareness sessions within school health programs and providing take-home materials for parents could improve knowledge and normalize screening; such initiatives have enhanced early cardiac detection elsewhere (29). Second, primary-care physicians should counsel parents using standardized Arabic leaflets that explain ECG goals, benefits, and data protection, as direct discussion by healthcare providers strongly increases acceptance (30). Third, offering free or subsidized ECG screening, particularly in public schools, would address the most frequently cited financial concern. Finally, coordinated public communication through school networks, social media, and endorsements by national cardiology societies and the Ministries of Health and Education would reinforce credibility and trust. Implementation and outcome evaluation of these measures could substantially strengthen parental understanding and ensure the sustainability of future ECG screening programs for children.

## Conclusion

Parental acceptance of ECG screening for school-aged children is influenced by factors such as awareness, education level, socioeconomic status, and concerns about privacy and medical follow-up. Increasing awareness through targeted educational initiatives and offering free or affordable screening programs are key strategies to promote broader acceptance.

## Data Availability

The raw data supporting the conclusions of this article will be made available by the authors, without undue reservation.
