# Supplementary material for: Parental acceptance of ECG screening for school-aged children: influencing factors and recommendations
Source: Front Public Health. 2025 Dec 17;13:1704354. doi: 10.3389/fpubh.2025.1704354 (PMC12753955; doi:10.3389/fpubh.2025.1704354)
Supplement: Supplementary file 1 [file Supplementary_file_1.docx]

Supplementary:

**Parental Education level and knowledge about inherited cardiac diseases**


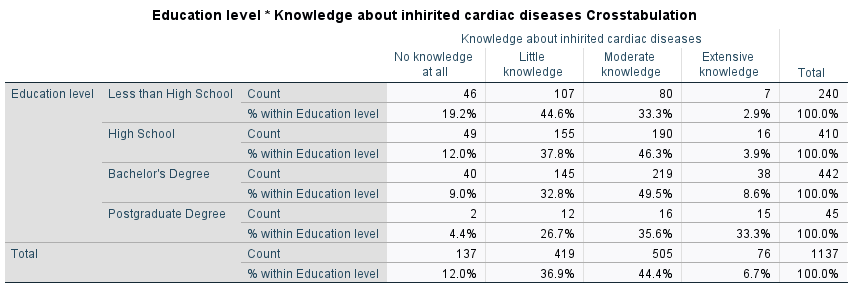


Figure S1 Parental Education level and knowledge about inherited cardiac diseases


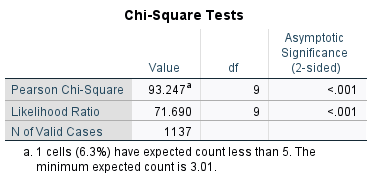


Figure S2 Higher education , higher knowledge

**Parental Level of education and knowledge about ECG**


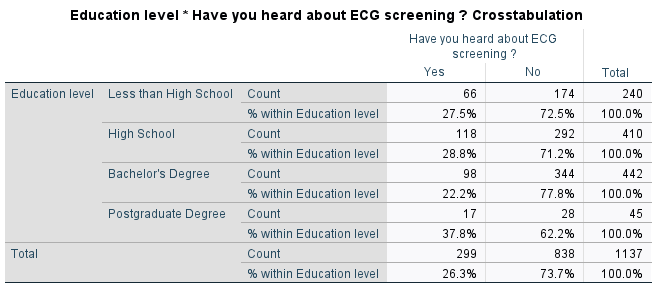


Figure S3 Parental Level of education and knowledge about ECG


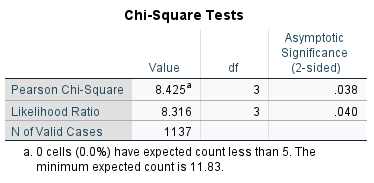


Figure S4 Higher education>> more knowledge about ECG

**Parental Knowledge and likelihood to approve ECG**


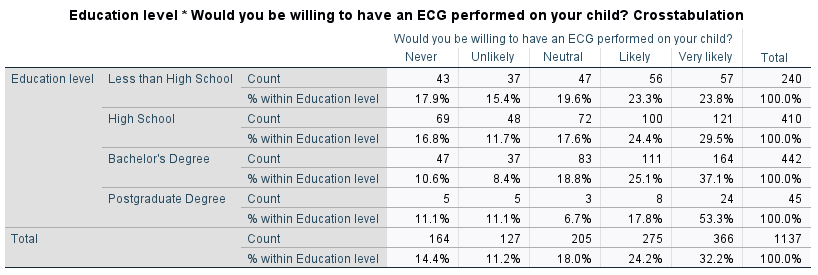


Figure S5 Parental Knowledge and likelihood to approve ECG


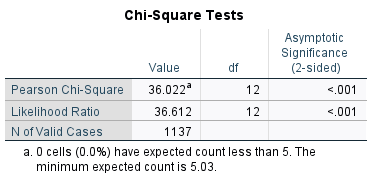


Figure S6 higher education >> more likely to approve, P<0.001

**Family history and knowledge about cardiac disease**


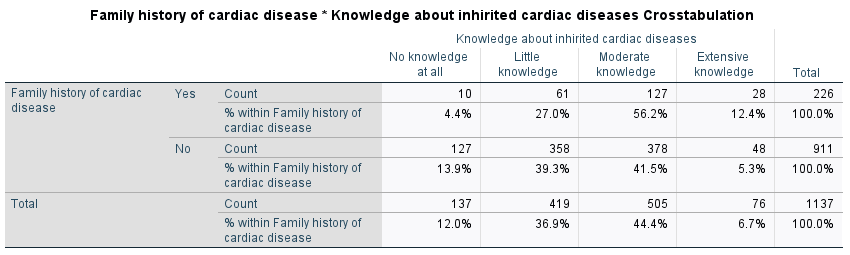


Figure S7 Family history and knowledge about cardiac disease


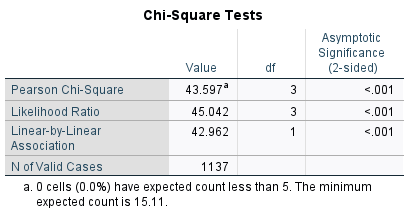


Figure S8 Positive family history >> higher percentage of good knowledge , p<0.001

**Family history and knowledge about ECG**


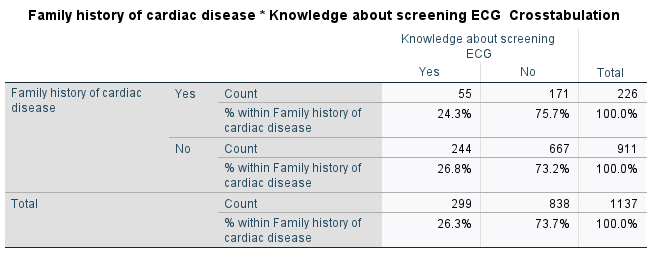


Figure S9 Family history and knowledge about ECG


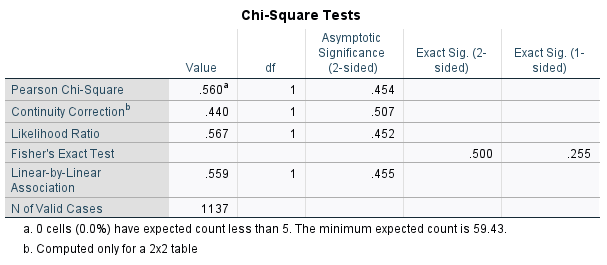


Figure S10 No association , p=0.454
